# Efficacy of Generic 2% Chlorhexidine Gluconate in 70% Alcohol Versus ChloraPrep for Preventing Surgical Site Infections in Orthopedic Surgery: A Scoping Review

**DOI:** 10.7759/cureus.94000

**Published:** 2025-10-07

**Authors:** Chigoziem A Ogbolu, Irrum Afzal, Syed S Ahmed

**Affiliations:** 1 Trauma and Orthopaedics, Lewisham and Greenwich NHS Trust, London, GBR; 2 Trauma and Orthopaedics, South West London Elective Orthopaedic, London, GBR; 3 Trauma and Orthopaedics, Tunbridge Wells Hospital, Tunbridge Wells, GBR

**Keywords:** chlorhexidine, cost-effectiveness, generic prep, orthopedics, skin antisepsis, surgical site infection

## Abstract

Surgical site infections (SSIs) are common in orthopedic procedures, increasing morbidity, mortality, and healthcare costs. ChloraPrep, which is branded 2% chlorhexidine gluconate in 70% isopropyl alcohol, is commonly used intraoperatively as a prepping agent due to its efficacy as an antiseptic agent. Generic/unbranded formulations of 2% chlorhexidine gluconate in 70% isopropyl alcohol have been demonstrated to be as effective as ChloraPrep across other surgical specialties, with the added benefit of lower costs. The aim of this scoping review article was to identify the available evidence base comparing the efficacy of generic/unbranded 2% chlorhexidine in alcohol with ChloraPrep in the prevention of SSIs in orthopedic surgical patients. This review has demonstrated a significant gap in the literature as to whether unbranded 2% chlorhexidine gluconate in 70% isopropyl alcohol is an efficacious alternative to ChloraPrep. Early evidence suggests that generic 2% chlorhexidine in 70% alcohol may effectively reduce infection and bacterial load in orthopedic surgery. Further rigorously designed randomized comparisons are needed to guide practice and procurement.

## Introduction and background

Surgical site infections (SSIs) remain a significant complication in orthopedic surgery. These postoperative soft tissue infections contribute to increased patient morbidity and mortality, prolong hospital stays, and impose substantial financial burdens on healthcare systems [1-3]. Reported postoperative infection rates in orthopedic patients vary widely, ranging from 1% to 13.2%. [4].

SSIs are most commonly caused by endogenous skin flora introduced into the surgical wound at the time of surgery [5,6]. Consequently, intraoperative skin antisepsis is a cornerstone of infection-prevention strategies. Several antiseptic formulations are available for this purpose, including chlorhexidine-based, povidone-iodine-based, and alcohol-based preparations [7]. Among these, 2% chlorhexidine in 70% alcohol has demonstrated superior efficacy in reducing both superficial and deep SSIs compared with other agents [3,8]. In recognition of this, the National Institute for Health and Care Excellence (NICE), which is a UK-based government-sponsored institution that provides evidence-based guidance and quality standards for healthcare, recommends such preparations as the first-line option for intraoperative skin antisepsis [9].

ChloraPrep, a widely used branded formulation containing 2% chlorhexidine in 70% alcohol supplied in a 26 mL applicator, has been extensively investigated and reported as an effective agent for intraoperative antisepsis in orthopedic surgery [10-13]. However, a major disadvantage of ChloraPrep is its high cost [13]. According to NICE, the price of a single 26 mL ChloraPrep applicator is £8.46, whereas generic alternatives such as Ecolab’s 2% chlorhexidine in 70% isopropyl alcohol (IPA) are considerably less expensive at £1.73 per 150 mL bottle [9]. Despite this cost disparity, there is limited evidence directly comparing the clinical effectiveness of generic/unbranded 2% chlorhexidine in alcohol with its branded equivalent. The NICE guideline committee has highlighted this gap and recommended further research into the comparative clinical and cost-effectiveness of different methods of application [9].

The primary aim of this scoping review was to identify and evaluate the available evidence comparing generic/unbranded 2% chlorhexidine in alcohol with ChloraPrep in the prevention of SSIs in orthopedic surgery. This review also sought to map gaps in the existing literature and provide direction for future research. The secondary aim was to assess whether any studies compared these preparations with respect to their ability to prevent SSIs or reduce bacterial colony-forming units (CFUs) at the surgical site, i.e., the number of viable bacterial cells.

## Review

Methods

This scoping review was conducted in accordance with the methodological framework proposed by Arksey and O’Malley [14]. This involves: identifying the research question, identifying relevant studies, study selection, charting data, summarizing, and reporting the results.

Literature Search

A comprehensive search of PubMed, EMBASE, MEDLINE, Cochrane Library, and Google Scholar was performed, covering the period from 1946 to April 2025. The search strategy was designed collaboratively by two authors (CO and IA) and adhered to the Preferred Reporting Items for Systematic Reviews and Meta-Analyses (PRISMA) guidelines. Search terms included combinations of the following: “Chlorhexidine” [MeSH], ChloraPrep [tw], “chlorhexidine gluconate in alcohol” [tw], “Surgical Wound Infection” [MeSH], “postoperative wound infection” [tw], “surgical wound infection” [tw], “Fractures, Bone” [MeSH], and ortho [tw]. The search was adapted for each database as required.

No restrictions were placed on publication date, language, location, or study design. Only human studies were included. Grey literature was explored using OrthoSearch, HMIC (Ovid), and Google Scholar. The reference lists of relevant studies were screened to identify additional eligible articles.

Study Selection

The initial search identified 457 articles, of which 276 remained after duplicates were removed. The strict inclusion criteria were research studies designed based on levels of evidence I-IV and including orthopedic patients undergoing surgery [15]. They also needed to evaluate the efficacy of generic/unbranded 2% chlorhexidine in 70% alcohol compared with ChloraPrep, with outcomes reported as either SSI within 30 days or bacterial colony-forming unit counts. Studies were excluded if they involved non-orthopedic surgical populations, assessed preoperative antisepsis techniques such as whole-body washes or baths, or investigated antiseptic agents other than chlorhexidine.

Titles and abstracts were independently screened by two authors, and disagreements were resolved through discussion. Studies that appeared relevant proceeded to full-text review, resulting in 137 articles being assessed in detail. Following full-text review, zero studies were identified that directly compared unbranded chlorhexidine in alcohol with ChloraPrep in orthopedic surgery.

**Figure 1:**
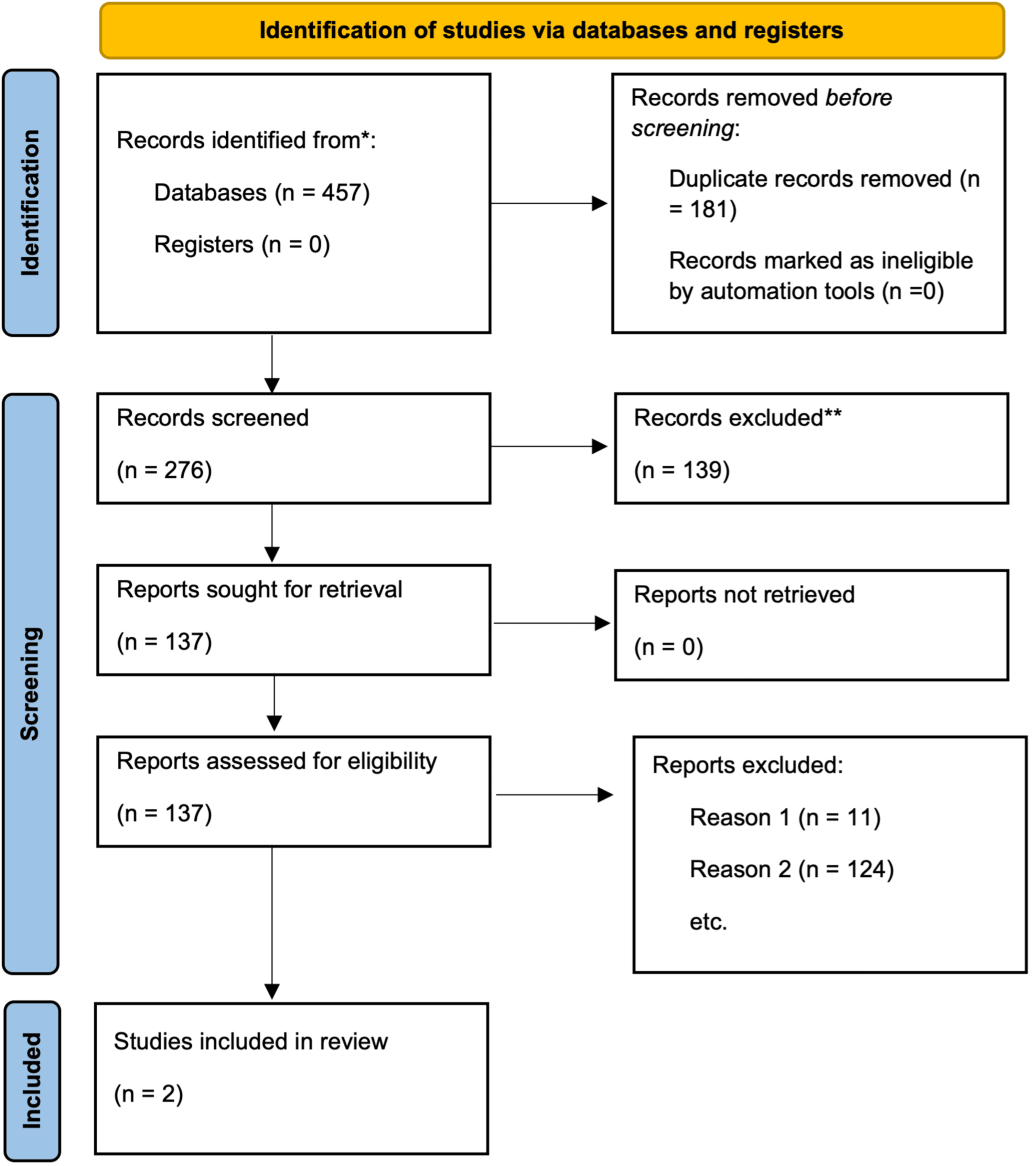
PRISMA flow diagram Flow diagram for study selection and searches of databases and registers [16]. Reason 1 Papers that reviewed preoperative antisepsis agents, reason 2 Papers that failed to compare generic prep and ChloraPrep intraoperatively

The study selection process is summarized in the PRISMA flowchart (Figure 1) [16].

Data Extraction

Data were extracted using a standardized form adapted from the Cochrane Collaboration framework. Information collected included author, year of publication, study design, country, surgical site, sample size, antisepsis preparation, and reported outcomes. Extraction was performed independently by CO and reviewed by IA, with no discrepancies arising during the process. It was agreed that any future disagreements would be resolved through consultation with the senior author (SA).

Results

The initial search yielded 457 articles, of which 276 remained after removal of duplicates. Following title and abstract screening, 137 studies were selected for full-text review. None of these met the predefined inclusion criteria of directly comparing generic or unbranded 2% chlorhexidine in 70% alcohol with ChloraPrep in orthopedic surgery.

Although no eligible comparative studies were identified, two relevant papers evaluating the use of unbranded 2% chlorhexidine in alcohol for orthopedic patients were considered for analysis in order to establish a preliminary evidence base. These studies are summarized in Table 1.

**Table 1:** Characteristics of studies included for review

Author	Year	Design	Country	Surgical Site	Participants	Preparation Method	Outcome Measured
Cao et al. [17]	2024	Cohort study	China	Foot and ankle	50	Gauze-soaked 2% chlorhexidine w/ 70% alcohol	SSI
Chang et al. [18]	2016	Randomized controlled study	Australia	Foot and ankle	51	Gauze-soaked 2% chlorhexidine in 70% alcohol	CFU count

Cao et al. (2024) investigated the efficacy of unbranded 2% chlorhexidine in 70% isopropyl alcohol for foot and ankle surgery using a two-step preparation method with gauze soaked in the antiseptic solution. Among 92 patients (mean age 40.6 years, mean BMI 24.3), three cases (6%) of superficial surgical site infection were reported at 12 months postoperatively, all successfully treated with oral antibiotics. No deep infections occurred. These findings suggest that unbranded chlorhexidine-alcohol solutions may provide effective antisepsis for clean orthopedic procedures, though the absence of a comparator group limits definitive conclusions.

Chang et al. (2016) evaluated bacterial reduction at the operative site using two application techniques: immersion of the limb in 100ml of unbranded 2% chlorhexidine in 70% alcohol via a plastic bag, and painting with gauze soaked in the same solution. Fifty-one patients were enrolled, with comparable demographic characteristics between groups. Both techniques achieved near-complete bacterial clearance (CFUs of 0 and 1, respectively), indicating strong antiseptic activity independent of the method of application.

Together, these studies provide preliminary evidence supporting the antimicrobial efficacy of unbranded 2% chlorhexidine in 70% isopropyl alcohol in orthopedic surgery. However, the lack of randomized or comparative studies highlights a significant gap in the literature and underscores the need for further research to establish clinical equivalence with branded preparations such as ChloraPrep®.

No studies directly comparing ChloraPrep with unbranded alternatives were identified. In accordance with the Arksey and O’Malley scoping framework, no formal risk of bias assessment was conducted. No formal quantitative analyses, such as meta-analysis or pooled effect estimates, were conducted, as no studies directly compared generic 2% chlorhexidine in alcohol with ChloraPrep in orthopedic surgery, making statistical synthesis methodologically inappropriate.

Discussion

Principal Findings

This scoping review highlights a critical gap in the literature: no studies were identified that directly compared the efficacy of unbranded 2% chlorhexidine in 70% alcohol with ChloraPrep in orthopedic patients. The absence of comparative evidence underscores the need for further research to evaluate whether unbranded preparations can demonstrate equivalent efficacy as an antisepsis agent at a substantially lower cost.

The two studies included in this review suggest that generic chlorhexidine in alcohol is effective at reducing superficial SSIs and bacterial load at the surgical site. Both employed application methods using gauze-soaked preparations, and both reported favorable outcomes. These findings suggest that generic products may represent a viable alternative to ChloraPrep; however, the available evidence is limited and lacks the power to establish equivalence or superiority.

Comparison with Existing Literature

The PREP-IT trial [19] and other large-scale studies [12,20] have demonstrated that ChloraPrep effectively reduces the risk of SSIs in orthopedic patients, supporting its widespread use. NICE similarly recommends chlorhexidine-alcohol solutions as first-line antisepsis, citing both clinical efficacy and cost savings achieved through infection prevention [9]. However, the primary drawback of ChloraPrep remains its high cost [21,22]. At £8.46 per 26 mL applicator, its use represents a considerable financial burden, particularly when compared with unbranded alternatives such as Ecolab, which costs £1.73 per 150 mL [9].

Evidence from outside orthopedics suggests that generic 2% chlorhexidine-alcohol solutions can be equally effective. For instance, Bibi et al. (2015) [23] reported reduced SSI rates in general surgery using unbranded chlorhexidine, while Dörfel et al. (2021) demonstrated bacterial load reduction in a controlled volunteer study [20,21,24]. Although these findings cannot be directly generalized to orthopedic surgery, they support the hypothesis that unbranded products may offer comparable clinical efficacy.

Implications for Practice

Current evidence suggests that generic chlorhexidine-alcohol solutions could be an effective, environmentally sustainable, and cost-efficient alternative to ChloraPrep [17,18]. Unlike disposable applicators, gauze-based methods allow for the use of sterilizable instruments, thereby reducing environmental impact. However, without direct comparative trials, widespread substitution cannot be recommended. NICE has emphasized the need for clarity regarding the method of application of chlorhexidine-alcohol solutions, and further evidence is required to inform both clinical practice and procurement policies [9].

Limitations

This review has several limitations. The absence of studies meeting the predefined inclusion criteria precludes any definitive conclusions regarding comparative efficacy. Only two relevant studies were included, both with small sample sizes (16-92 participants). Furthermore, both focused exclusively on foot and ankle surgery, restricting applicability to other orthopedic subspecialties. Variation in antiseptic application methods and outcome measures (SSI vs. CFU count) complicates cross-study comparison. Additionally, neither study directly compared generic chlorhexidine with ChloraPrep, and no randomized controlled trials were identified. These limitations underscore the inadequacy of evidence available and highlight the urgent need for high-quality comparative research.

## Conclusions

This scoping review demonstrates a substantial gap in the literature regarding the comparative effectiveness of generic/unbranded 2% chlorhexidine in 70% alcohol and ChloraPrep in preventing SSIs in orthopedic surgery. While limited evidence indicates that generic preparations may reduce infection rates and bacterial burden, the current evidence base is insufficient to guide clinical practice or procurement policy.

Future research should focus on well-designed, multicenter randomized controlled trials directly comparing ChloraPrep with generic chlorhexidine-alcohol preparations. Such studies should incorporate clinically relevant outcomes, including SSI rates, long-term follow-up, and cost-effectiveness analyses. Environmental impact assessments should also be considered, given the potential sustainability benefits of reusable application methods. Generating robust evidence in these areas will be essential to inform cost-conscious, evidence-based decision-making in orthopedic surgical practice.
